# Amarula Waste to Activated Carbon: A Low-Cost Adsorbent for Deep Desulphurization of Model Diesel Fuel

**DOI:** 10.1021/acsomega.5c08670

**Published:** 2026-07-29

**Authors:** Tsepiso Kabi, Liberty L. Mguni, Joshua Gorimbo, Yue Zhao, Xiaojin Gao, Haipeng Xu, Ryno van der Merwe, Yali Yao

**Affiliations:** † Institute for Catalysis and Energy Solutions, College of Science, Engineering and Technology, University of South Africa, Johannesburg 1710, South Africa; ‡ Science and Technology Service Platform, 12689 Qilu University of Technology (Shandong Academy of Sciences), Jinan 250014, China; § South African Nuclear Energy Corporation SOC Ltd. (Necsa), Pretoria 0240, North West Province, South Africa

## Abstract

This study aimed to address two significant environmental challenges: waste management and climate change mitigation, by converting biomass waste from Amarula liqueur production into activated carbon (AC) for adsorptive desulphurization. Biomass from Amarula fruit, seed, and shell waste was subjected to pyrolysis at 800 °C and steam activation (45 min), producing activated carbon with surface areas of 740, 437, and 245 m^2^/g, and pore volumes of 0.37, 0.21, and 0.17 cm^3^/g, respectively. Furthermore, shell-derived carbon activated for 90 min achieved a significantly higher surface area of 1194 m^2^/g and a pore volume of 0.98 cm^3^/g with commercial prospects. The activated carbons were evaluated for their ability to remove dibenzothiophene (DBT) from a model diesel fuel in a batch reactor. Desulfurization efficiencies of 65 ± 4, 54 ± 3, and 31 ± 6% were achieved for the fruit-, seed-, and shell-derived carbons, respectively. Notably, the shell-based activated carbon produced with extended steam exposure exhibited the highest adsorption efficiency of 73 ± 3% for model fuel. However, its poor activity for conventional diesel was attributed to the poor selectivity of the adsorbent. The desulfurization performance was found to be strongly influenced by the presence of surface O-functional groups and the adsorbents’ textural properties. In conclusion, the study demonstrates that Amarula biomass waste can be valorised into effective activated carbons for cleaner fuel applications, though further optimization is needed to enhance selectivity and extend their applicability across a wider range of fuel systems.

## Graphical abstract

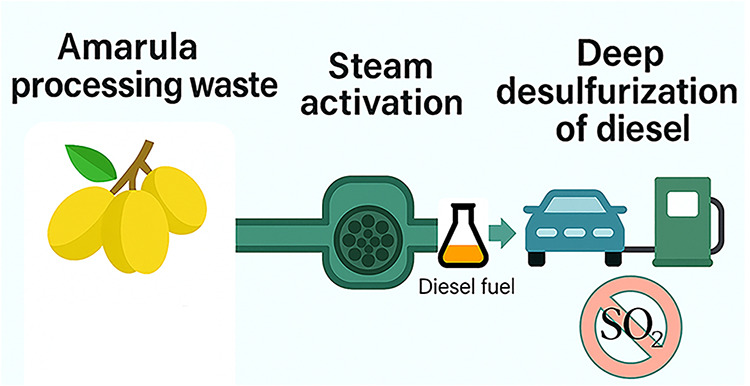

## 1 Introduction

The marula (*Sclerocarya birrea*) fruit is indigenous to Africa. The marula trees are now cultivated for industrial purposes to produce large quantities of the worldwide-known “Amarula” spirit, with approximately 1.2 million 9 L cases sold per year.^1^ A large amount of waste is produced during the production of the Amarula liqueur. This Amarula waste consists of low-quality fruit residues and seed waste generated during pulp extraction. These wastes are discarded and dumped into a landfill, where they rot and decompose, producing harmful gases that contribute to environmental pollution.^2^ For these reasons, this study chose to convert Amarula waste biomass (fruits, seeds, and shells) into a valuable product, activated carbon (AC), to develop a process that helps mitigate waste management issues.

Activated carbons are carbonaceous materials that have been processed to create materials with various pore sizes and high-surface areas. They consist mainly of cyclic carbons bonded to each other by sp^2^ and sp^3^ hybridization, with heteroatoms on the surface. Depending on the type of processing, the AC produced may be either graphitic or nongraphitic.^3,4^ Different carbonaceous materials produce AC with distinct characteristics. Marsh and Rodriguez-Reinoso^3^ and Savova et al.^5^ used olive stones, cherry stones, apricot stones, and almond shells to produce AC under the same conditions. They found that a precursor plays an important role in the textural properties. Lo et al.^6^ used Ma bamboo and Moso bamboo as precursors for ACs and found that the ACs of these bamboos had different characteristics despite their exact environment. The production of ACs involves microwave and conventional heating treatment. Researchers^7–10^ compared AC production using both microwave and conventional heating methods and found that although the latter is time-consuming and energy-intensive, it is widely preferred for large-scale production. In this study, the latter treatment has been used because it is suitable for scaling-up purposes.

It has also been reported that the properties of AC depend on the activation methods used for processing. Therefore, the production of AC involves either a single-step or a two-step synthesis process: carbonization or pyrolysis under inert conditions, followed by activation at an elevated temperature. The activation process can occur by chemical activation,^7,11–23^ using chemicals such as KOH, K_2_CO_3_, HNO_3_, H_2_SO_4_, H_3_PO_4_, or ZnCl_2_; or by physical activation^3,16,24–29^ using steam, CO_2_, or O_2_; or by combining both chemical and physical activation^30,31^ in one process. Some researchers^32–36^ compared the properties of AC produced from steam and CO_2_. They found that the activated carbons from steam had wider micro- and mesopores, whereas the AC from CO_2_ had narrower micropores. Other researchers found that as steam increases, micropores widen, mesopore volume decreases, and surface area decreases.^16,37,38^ It has also been reported that increasing the activation temperature increases surface area and pore volume.^38,39^ Some researchers^29,40–42^ reported that higher temperatures ruptured and destroyed the surface area, creating more macropores but fewer micropores. Moreover, the material became more graphitic.

Several researchers^3,25,43^ have studied the mechanism of steam activation. The steam oxidizes carbon (C) from a carbonaceous solid to form a water–gas product consisting of carbon monoxide (CO) and hydrogen (H_2_), as shown in [Disp-formula rea1] and [Disp-formula rea2] below. Hydrogen gas then burns to form a water (H_2_O) molecule, which further oxidizes CO to form CO_2_. The CO_2_ extracts carbon from its structure and oxidizes it to CO ([Disp-formula rea3]), forming more meso- and macropores.

R1 $$C+H_{2}O⇌\mathrm{CO}+H_{2}\Delta H=117\;\mathrm{KJ}/\mathrm{mol}$$

R2 $$\mathrm{CO}+H_{2}O⇌{\mathrm{CO}}_{2}+H_{2}\Delta H=-41\;\mathrm{KJ}/\mathrm{mol}$$

R3 $$C+{\mathrm{CO}}_{2}⇌\mathrm{CO}\Delta H=171\;\mathrm{KJ}/\mathrm{mol}$$

Diesel fuel is a distillate of crude oil. It contains toxic compounds such as organosulfur compounds, dibenzothiophene (DBT), and its alkyl derivatives. These sulfur compounds have a negative impact on the environment (agriculture and global warming), the economy (corrosion), and human health (respiratory infections). Moreover, they are difficult to remove using technologies such as hydrodesulfurization (HDS).^44^ Adsorptive desulfurization (ADS) has been proposed as a promising supplement to HDS because it can be carried out at ambient conditions, addressing SO*_X_*, carbon, and energy concerns simultaneously. Moreover, ADS can remove sterically hindered alkyl organosulfur compounds that are difficult to reduce using other techniques, such as HDS. A number of activated carbon sources have been reported, including pine wood,^45^ coconut,^46^ groundnut shell,^47^ waste tires,^48^ coal,^49^ Brewer’s spent grains,^50^ sucrose,^51^ and polystyrene.^52^ The quality of the produced AC is greatly dependent on the source material, as discussed earlier. To the best of our knowledge, no work has been reported on activated carbon from the Amarula waste.

This study aims to address the environmental and economic challenges associated with Amarula waste management by converting it into high-surface-area activated carbon for the deep desulphurization of diesel fuel. By developing a cost-effective and sustainable adsorbent from locally available waste materials, this research seeks to mitigate pollution, reduce waste, and improve the efficiency of diesel desulphurization processes.

## 2 Materials and Methodology

AC was prepared by using a two-step synthesis process that involved pyrolysis and steam activation. The desulphurization process was carried out using a batch reactor.

### 2.1 Materials

The biomass used was Amarula waste (fruit, seeds, and shells). The following reagents and equipment were used: horizontal electric tube furnace, quartz tubes, quartz crucible boats, Afrox nitrogen gas (99% purity), chemical (99% pure reagents from Sigma-Aldrich), dibenzothiophene (DBT), hexadecane, and toluene.

### 2.2 Sampling and Processing of Amarula Waste Biomass

The Amarula waste used as biomass was collected in Phalaborwa, Limpopo province, South Africa. The Amarula waste samples collected were divided into three categories: Amarula fruit (AmWa), seeds (AmSe), and shells (AmSh). Each part was used to produce AC under the same conditions by using pyrolysis and steam activation. In our previous work, amSh waste biomass was used to determine the effects of applying pyrolysis temperatures (600, 700, and 800 °C) on biochar.^53^ A temperature of 800 °C was selected as the best pyrolysis temperature for biochar. The pyrolysis of Amarula waste biomass was carried out by weighing ≈3 g of dried AmSh waste into each quartz boat crucible, and three boats were used per run. The samples were placed inside a quartz tube and then placed in a horizontal furnace. Nitrogen gas (N_2_) was flowed at 60 mL/min for 10 min before the temperature was raised to 800 °C at a ramping rate of 10 °C/min for a residence time of 2 h. At the end of the process, the system was cooled to room temperature, and the biochar yield was determined. The pyrolyzed biochar was then stored for later use in a tightly sealed container after being labeled AmSh-B-800, “B” denotes biochar, and “800” denotes the pyrolysis temperature. The AmSe and the AmWa waste samples were pyrolyzed as described above and named AmSe-B-800 and AmWa-B-800, respectively.

The effect of steam residence time was recorded at 30, 45, 60, 75, and 90 min intervals when using the AmSh biochar. A steam residence time of 45 min was selected and used for the other Amarula waste samples. The steam activation was carried out by crushing biochar AmSh-B-800 (>3 mm), weighing it first into a crucible, and then placing it in a horizontal tube furnace. Thermal energy was applied at a rate of 10–800 °C under the N_2_ flow rate of 60 mL/min. The supply of inert gas (N_2_) was stopped when the set temperature was reached. The activating gas (steam) temperature was kept constant at 120 °C by using a temperature controller connected to a magnetic-stirring heating mantle. The steam was fed into a horizontal reactor at a rate of 5 mL/min for 45 min. The steam supply was stopped, and the N_2_ flow resumed until the horizontal reactor cooled to room temperature. The steam-AC produced was weighed to determine the percentage yield and the percentage burnoff, then stored in a tightly sealed container, and labeled AmShAC-800ST-45 min for the AmSh waste; AmSeAC-800ST-45 min for the AmSe waste; and AmWaAC-800ST-45 min for the AmWa waste.

### 2.3 Characterization of Processed Amarula Waste Biomass

#### 2.3.1 Thermal Analysis of Processed Amarula Waste Biomass

An AC with a higher percentage burnoff tends to have higher activity than one with a lower percentage burnoff. For this reason, ACs were characterized by calculating the percentage yield, as shown in eq 1.

1 $$\%\;\mathrm{yield}=\left(m_{i}-m_{f}\right)/m_{i}\times 100$$

where *m*_i_ is the initial mass before thermal treatment; *m*_f_ is the final mass after steam activation.

The percentage burnoff characterizes the mass loss of carbon during pyrolysis or activation. Proximate analysis was performed using the Thermal Gravimetry Analysis (TGA) technique in an air atmosphere at a flow rate of 60 mL/min using the TGA5500-0026 model. The process temperature was ramped from 50 to 800 °C at 10 °C/min. Platinum HT pans were used as sample holders. Derivative thermogravimetry (DTG) analysis was also performed under the same conditions.

#### 2.3.2 Microscopic Analysis

Micrographs of processed Amarula waste were obtained using field-emission scanning electron microscopy (FESEM) JEOL JSM-7800F, coupled with energy-dispersive X-ray spectroscopy (EDS). Before analysis, the samples were coated with gold using a sputter coater. Transmission electron microscopy (TEM) analysis was performed on selected AmSh waste ACs; the ACs were dispersed in an ethanol solution and then sonicated for 15 min. A drop of the sample’s suspension was then placed onto a copper grid. This was subjected to the TEM technique using a JEOL 2010F equipped with a Schottky field-emission gun and with an ultrahigh-resolution pole piece providing a point resolution of 1.9 Å.

#### 2.3.3 Spectroscopic Analysis

Previously dried potassium bromide (KBr) was mixed with each thermally processed Amarula waste biomass sample to make pellets in a ratio of 1:100. Pelleted AC samples were subjected to the Fourier transform infrared (FTIR) spectroscopic technique using the PerkinElmer Frontier model. The analysis of the functional groups present was carried out at resolutions of 8 and 16 cm^–1^, with scans spanning 4000–400 cm^–1^. The X-ray diffraction patterns were determined using Bruker’s D2 Phaser X-ray diffractometer using Cu–Kα (=0.15406 Å). The radiation source was operated at 40 kV and 25 mA. The diffraction angle (2θ) was varied from 10 to 90° using a scan rate of 4.2 °C/min. Peak analysis was carried out using Origin Pro 8.5 software, and peak deconvolution was performed using Gaussian fitting. The lattice (*L*) size for crystalline (*L*_c_) and amorphous (*L*_a_) was further determined using Scherrer eq 2

2 β=Kλ/L⁡cos⁡θ

where *K* is the constant, for which 0.9 was for *L*_c_, and 1.84 was for *L*_a_; γ is the wavelength; β is the full width at half-maximum (FWHM); and θ is the angle of the observed peak. The interlayer spacing *d*_002_ was carried out using eq 3

3 $$d_{002}=\lambda /2\sin\theta$$

#### 2.3.4 Textural Properties

Brunauer–Emmett–Teller (BET) surface area and porosity analyses were carried out using a Micrometrics TriStar II3020 under N_2_ adsorption at −196 °C. The samples were first degassed for 12 h at 160 °C under an inert gas flow. Micropore and mesopore distributions were evaluated using Barrett–Joyner–Halenda (BJH).

### 2.4 Application to the Desulphurization of Model Diesel DBT

#### 2.4.1 Preparation of ≈100 ppm DBT Model Diesel

About 100 ppm dibenzothiophene (DBT) model diesel fuel was prepared by dissolving it in a solvent mixture of 85% hexadecane and 15% toluene. All of the analyses of model diesel were carried out using the gas chromatography-pulse flame photometric detector (GC-PFPD) technique. The desulphurization process is summarized in Figure 1.

**Figure 1:**
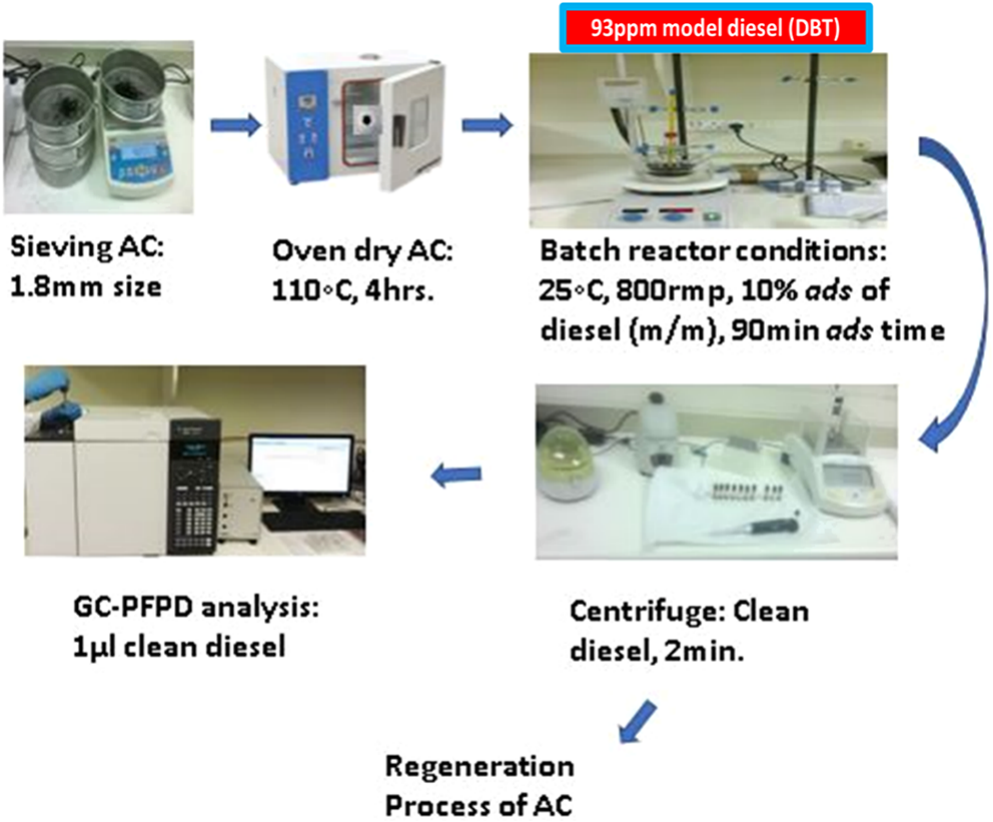
Schematic of the desulfurization process of DBT model diesel fuel, using processed Amarula waste.

#### 2.4.2 Desulphurization of Diesel Fuel (DBT)

The synthesized AC from AmSh waste was sieved to obtain particles of 1–2 mm. The sieved AmShAC-800ST was then dried in an oven at 110 °C for 4 h. The dried AC was then weighed into a 50 mL round-bottomed flask using an analytical balance, and its mass was recorded. Then, 10 mL of model diesel (DBT) was added to the flask containing the weighed AC. The reactor (flask) was immersed in a water bath at 27 ± 2 °C with continuous stirring at 800 rpm of a magnetic stirrer. Samples of 100 μL were taken and pipetted into the 0.5 mL centrifuge tubes at predetermined time intervals within 180 min. The samples were centrifuged for 5 min. Then, 1 μL of the centrifuged clean model diesel was injected into a GC-PFPD for analysis. The content of organosulfur in the model diesel was determined from the chromatograms obtained. The adsorption efficiency percentage and adsorption capacity were calculated using eqs 4 and 5, respectively. The *C*_0_ and *C_t_* are the concentrations of DBT in the model diesel at the initial time and at time *t* in ppm, respectively. The *q_t_* was the adsorption capacity at time *t* in mg/g; *W*_soln_ and *W*_ads_ are the masses of the DBT model diesel solution and processed Amarula waste, respectively.

4 $$\mathrm{de}\mathrm{sulphurization efficiency}\;\%=\left(\left(C_{0}-C_{t}\right)/C_{0}\right)\times 100$$

5 $$q_{t}=\left(C_{0}-C_{t}\right)W_{\mathrm{soln}}/W_{\mathrm{ads}}$$

Adsorptive desulfurization of real diesel was carried out under the same conditions. The amount of sulfur in the diesel was determined using eq 6, with DBT as the reference compound at 100 ppm.^54^

6 $$C_{s,i}=C_{s,\mathrm{ref}}\frac{{A_{i}}^{0.5}}{{A_{\mathrm{ref}}}^{0.5}}$$

The recycling experiments were carried out by refluxing the used adsorbents in octane for 3 h, followed by drying them in an oven at 110 °C overnight.

## 3 Results and Discussion

### 3.1 Characterization

#### 3.1.1 Percentage Burnoff for the Processed Amarula Waste

Figure 2a shows the effect of steam residence time on the percentage burnoff for AmShAC. The results show that the percentage burnoff increased with increasing steam residence time. This was because the longer the carbon is exposed to water molecules, the more the active carbon layers are extracted from the solid carbon, and the more the pores widen. Other researchers^37,55,56^ also reported the trend. Bergna et al.^25^ reported similar results when they produced AC using steam at 800 °C. At a holding time of 60 min, a yield of 34.3% was achieved. But when using 240 min as the holding time, they obtained a lower percentage yield. In 2018, the research team of Bergna et al.^57^ used wood chips to produce ACs with steam residence times of 120 and 240 min, respectively, and found similar trends in percentage yield.

**Figure 2:**
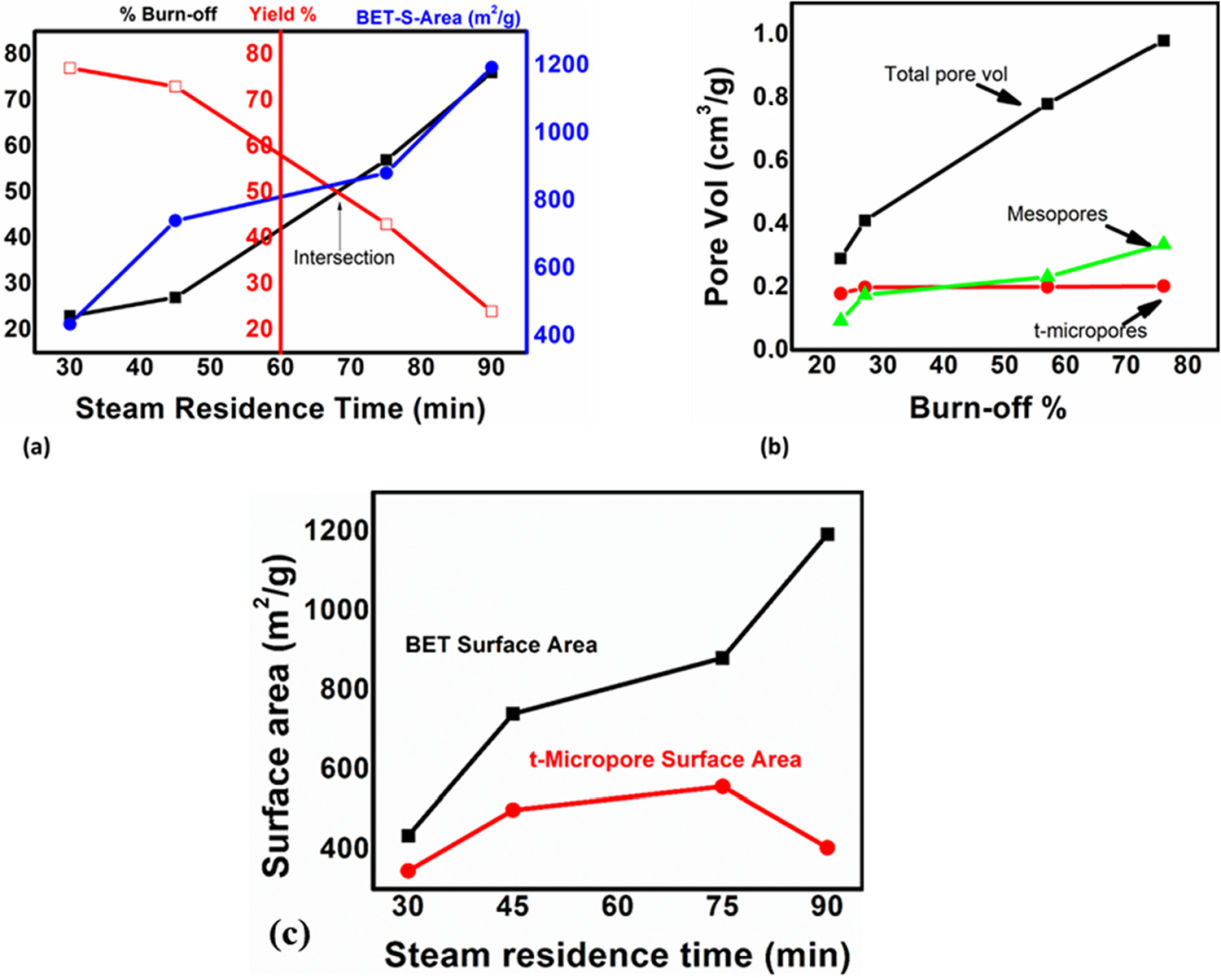
Effect of steam residence time on (a) percentage burnoff, (b) porosity, (c) t-micropore surface area, and BET surface area for AmShAC.

In this study, using AmSh waste as a precursor showed that a 50% burnoff could be obtained at a 70 min residence time and a temperature of 800 °C, as indicated by the intersection point (Figure 2a). This would be ideal for industrial applications or for scaling up to balance both yield and burnoff percentage without compromising product quality.

Figure 2b shows that as steam residence time increased, the porosity of the AmSh ACs increased. The graph also shows that more mesopores were formed above 50% burnoff. This is contrary to the findings obtained by Rodríguez-Reinoso et al.;^35^ the volume of the mesopores and macropores decreased at a higher percentage of burnoff. The nature and composition of the waste material used were different from those of our material. Hence, different feedstocks can lead to variations in pore structure development during activation. Other researchers^58,59^ found that BET surface area and pore volume decreased as steam residence time increased. For this reason, further textural analysis was done to determine the t-micropore surface area of Amarula waste AC. It was found that the t-micropore surface area increased with steam residence time up to a peak at 75 min, as shown in Figure 2c. The micropore surface area then decreased as the steam residence time increased further to 90 min. This was because the existing micropore surface area ruptured as more steam widened and deepened available micropores. The effect was a reduction in the surface area of the micropores. Therefore, this study found that the micropore surface area “decreased” at some point as the steam residence time increased, whereas the BET surface area increased with increasing steam residence time.

Upon comparing the percentage burnoff for different Amarula waste ACs (Table 1), it was found that the AmWa waste had a higher percentage burnoff, followed by the AmSh waste and then the AmSe waste ACs. This trend can be attributed to variations in the constituents of the precursor materials. The AmWa waste ACs precursor had edible pulp and a nut inside the shells. The Amarula seed waste AC precursor had only the nuts inside the shell, whereas the shell waste AC had the pulp, but the nuts were removed. Savova et al.^5^ also reported variations in percentage burnoff when they produced ACs from almond shells, nut shells, apricot stones, cherry stones, and grape seeds.

**Table 1:** Percentage Burnoff for Three Types of Amarula Waste ACs at 45 min Residence Time, 800 °C

AC type	% burnoff	% yield
AmShAC-ST	27 ± 4	73 ± 2
AmSeAC-ST	25 ± 3	75 ± 3
AmWaAC-ST	30 ± 5	69 ± 4

#### 3.1.2 Thermal Analysis of Processed Amarula Waste

Several researchers have applied thermal analysis to determine the decomposition stages of materials.^24,27,40^ In this study, TGA was performed on the processed Amarula waste (AmSeAC-ST, AmSeAC-ST, and AmWaAC-ST), which showed three decomposition stages (Figure 3). The first stage (<100 °C) was due to dehydration. It showed a tiny curve, signifying less moisture content on the surface of processed Amarula waste than unprocessed Amarula waste.^53^ The second stage, between 400 and 600 °C, was due to devolatilization. It was caused by the thermal decomposition of oxygen groups on the surface of ACs and during the cracking and deformation of C=C and C–H in the aromatic ring of AC.^60^ The third stage, between 600 and 800 °C, was due to the retention of residue products that consisted of inorganic matter and fixed carbon.

**Figure 3:**
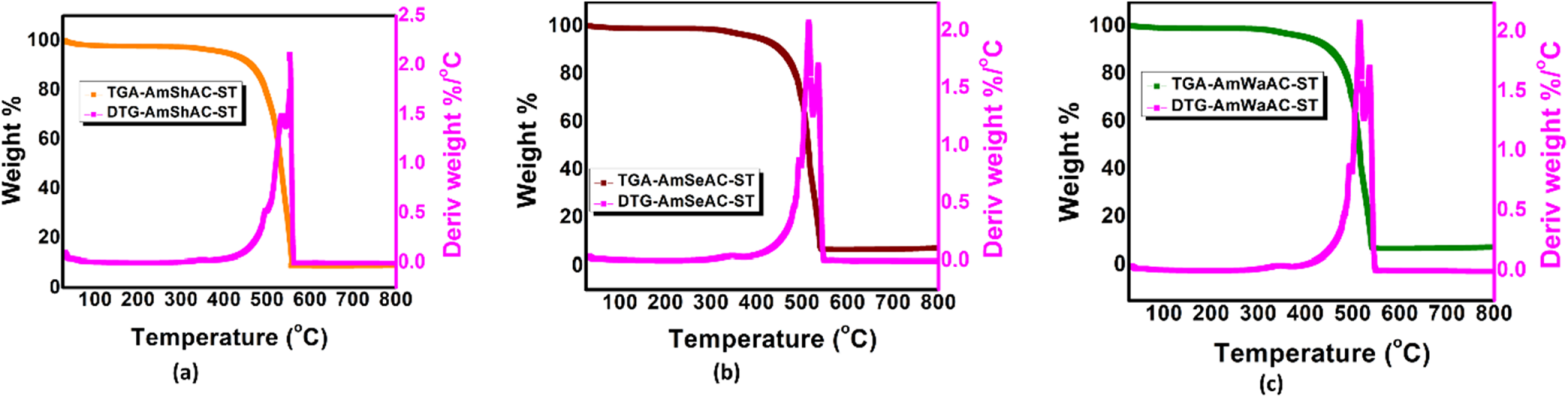
TGA/DTG graph under an air atmosphere for Amarula waste ACs: (a) AmShAC-ST, (b) AmSeAC-ST, and (c) AmWaAC-ST.

The total mass loss during thermal analysis was found to be 91.7, 93.6, and 98.8% for AmShAC-ST, AmSeAC-ST, and AmWaAC-ST, respectively. Despite the waste being from the same plant, the differences in mass loss confirm that the precursor of AC does play an important role in the thermal characteristics of the final product, as stated by other researchers.^37,55,56^ It was further found that the processed Amarula waste was more thermally stable than the unprocessed Amarula waste from our previous work.^53^ This was due to the strong bonds formed between carbon and carbon atoms during thermal processing. This formation of strong bonds might have occurred because of the breakdown of volatiles, leaving only a carbon structure, which is single-bonded to one another by sigma bonds. Other researchers obtained a similar TGA curve when analyzing ACs derived from biomass.^40^

The peak temperatures for each Amarula waste AC from the DTG curve are shown in Figure 3. The three identified peaks in each graph show the amount of evolved volatiles, such as CO_2_, CO, and CH_4_, during thermal decomposition of volatiles on ACs.^60^ It was further observed that the last peak of Amarula shell ACs occurred at a much higher temperature than that of AmSe and AmWa waste ACs. This confirms the ability of AmShAC-ST to be more resistant to thermal treatment than the other two samples. Even though the Amarula waste ACs can be used as an adsorbent at room temperature, the TGA/DTG results show that there is great potential in this type of waste for use as a catalyst or catalytic support for processes that occur at temperatures of ≈500 °C.

#### 3.1.3 SEM-EDX Analysis

The morphology of the Amarula waste ACs is shown in Figure 4. It reveals the presence of cavities and large pores on the surface, unlike in the unprocessed Amarula waste biomass, see ref 53, which showed only a smooth surface with no observable pores. Other researchers also observed cavities and large pores on ACs.^33,61^ The formation of the observed pores is due to the elimination of volatiles from the biomass during the first stage of thermal processing­(pyrolysis). This process leaves only a carbon material, called biochar. The application of steam to biochar tends to widen the pores depending on the source type. This process occurs because every time a layer of carbon is removed, a hole or pore is formed. The removed carbon dissipates into the environment as carbon monoxide/carbon dioxide formed by the reaction with oxygen in the water molecule.

**Figure 4:**
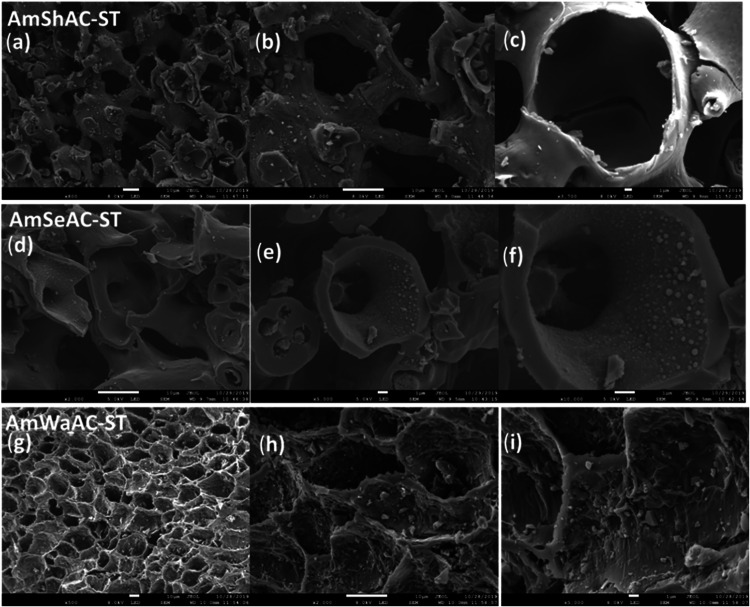
SEM images of Amarula waste ACs at different magnifications: Images (a–c) are for AmShAC-ST; (d–f) are for AmSeAC-ST; and (g–i) are for AmWaAC-ST.

The Amarula shell waste processed to AC showed a network of holes and tiny particles on the surface and large, deep open pores in a network format (Figure 4a). Further magnification (Figure 4b,c) revealed large, irregular semiopen pores with smooth walls and observable cracks inside the pores. This micrograph resembles a glassy carbon image observed by O’Malley et al.^62^ According to the model, the AC structure is a tangle of carbon-atom threads arranged into different ring structures. The spaces between the threads result in various pore sizes, making adsorbing molecules of different sizes easy.^63^ On the other hand, the morphology of Amarula seed ACs (Figure 4d) has a smoother surface compared to that of the Amarula shell AC, and the pores have a cone-like shape with smooth edges. Further magnification (Figure 4e,f) of the SEM image revealed tiny spheres inside the walls of the pores. The pores’ spheres were similar to the carbon aerogel structure modeled.^64,65^ The spheres in AmSeAC appeared naturally; according to Gavalda et al.,^65^ the structure of the spheres depicts the graphitic microcrystalline with some layers, which contribute further to the heterogeneous adsorption process. The spherical morphology was also seen on other synthesized carbon materials.^66–68^ Furthermore, the images reveal irregular semiopen pores with smooth internal walls and a tangled network of fibrous carbon threads, formed during steam activation as volatile species escaped and carbon layers restructured.

The AmWa waste AC (Figure 4g) showed a cage-like morphology with a rough surface and pronounced wall roughness within the pores. The pores appeared to be wider but did not extend deep to the end (Figure 4h,i), unlike the AmSe and the AmSh waste ACs. This type of morphology resembles the carbon structure first described by Byrne and Marsh,^63^ which is characteristic of low-density carbon. This structure exhibits slit-like pores, leading to heterogeneous adsorption sites.

SEM images revealed fine particulate deposits on the surfaces of the Amarula-waste-derived activated carbons. Elemental identification by EDX (Table 2) confirmed that these particles originate from inorganic constituents, such as potassium, magnesium, calcium, and silica. The presence of mineral ash was further corroborated by TGA analysis, which showed residual inorganic matter remaining after thermal treatment.

**Table 2:** Elements Identified by EDX for Processed Amarula Waste

EDX analysis of processed Amarula wastes						
AmShAC-ST			AmSeAC-ST		AmWaAC-ST	
elements	element wt %	atom %	element wt %	atom %	element wt %	atom %
C	78.4	97.0	68.0	86.2	59.9	84.2
O	0.8	0.7	3.5	3.3	8.1	8.6
N			7.7	8.4		
K	0.9	0.4	1.7	0.7	9.5	4.1
Mg	0.3	0.2			0.6	0.4
Ca	1.0	0.4				
Si					1.5	0.9

Multiple regions of the carbon surface were examined by EDX, and the results showed only minor compositional variations across different spots, indicating a relatively uniform distribution of these inorganic species. The results presented in Table 2 correspond to the full-area EDX scan, providing an overall representation of the elemental composition.

#### 3.1.4 TEM Analysis

TEM analysis was carried out on the selected AmSh ACs, as shown in Figure 5. The observed dark parts in Figure 5a signify amorphous carbon or graphitic, whereas the bright parts were considered to be pores, confirming the SEM analysis above. The holes observed in the magnified images in Figure 5b,c were caused by the tangling of fibers, as observed in SEM micrographs. Therefore, these images confirm that a porous AC was indeed synthesized from AmSh waste biomass. Other researchers observed similar images.^69–71^

**Figure 5:**
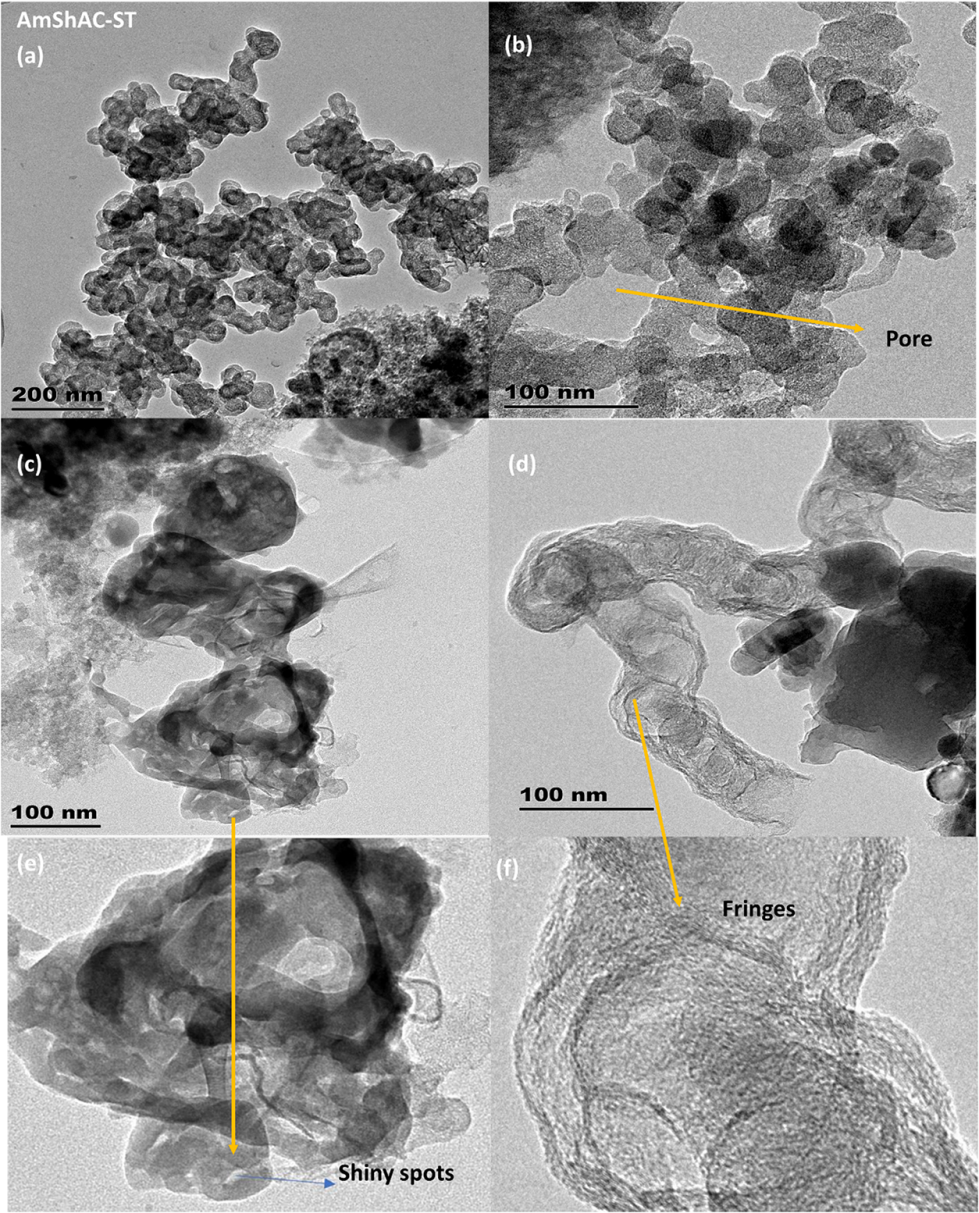
TEM micrographs of AmShAC-ST at different magnifications showing structural features: (a) porous carbon matrix; (b, c) magnified images showing pore formation; (d) hollow-ring fiber morphology characteristic of amorphous carbon; (e) bright crystalline-like particles attributed to diamond-like carbon domains; (f) graphitic lattice fringes.

The structure in Figure 5d looked like a hollow-ring fiber, which represents an amorphous carbon observed in a 2θ value of 23°. The morphology in Figure 5e further showed some white dots that are more like shiny particles in the structure of AC, as in the TEM image.^72^ This may be attributed to the geometry of the diamond structure. This shiny structure may be attributed to another carbon type that is crystalline in AC, as confirmed by the XRD at the 2θ value 44°. The fringes observed in Figure 5f confirm the stickering of carbon layers for 5-, 6-, or 7-membered rings in the structure of AmSh waste AC.^33,69,73–75^ The TEM micrographs further show graphitic fringes and ring-like carbon arrangements, confirming the presence of partially ordered sp^2^ carbon domains consistent with the XRD results. The tiny spherical features observed within the pores are likely mineral residues or carbon nanoparticles originating from inherent inorganic species (K, Ca, Si) in the Amarula waste, which may have catalyzed localized graphitization. These morphological features collectively indicate that processed Amarula waste-derived AC possesses a hierarchical porous structure with partially crystalline graphitic regions.

#### 3.1.5 XRD Analysis of Amarula Waste ACs

The XRD analysis of unprocessed Amarula waste biomass in our previous work showed a highly intense peak at the 002 plane due to crystalline cellulose and hemicellulose.^53^ The thermal processing of these Amarula waste samples broke down cellulose and hemicellulose, reducing the intensity of the (002) reflection peak and broadening it. This was due to a decrease in the order of crystallinity as a result of heat treatment. The partial graphitization occurs at high activation temperatures (800 °C), and carbon layers undergo structural rearrangement and stacking into short-range graphitic microcrystals. Additionally, the steam-activation process enhances this ordering by promoting localized carbon–oxygen gasification reactions, while the inherent mineral content (e.g., K, Ca, Fe, and Si) in the biomass may catalyze graphitic domain formation. This consequently shifted the asymmetric bands of 002 to 2θ angles valued at 22.9, 23.2, and 24.7° for AmSh, AmSe, and AmWa waste ACs, respectively, as shown in Figure 6a. The second weak broad peak was observed at approximately a 2θ value at 44° with a basal reflection plane of (100). This peak was formed in response to the high randomness in the processed Amarula wastes. It denotes the presence of amorphous carbon.^74,76–79^ For these reasons, the Scherrer expression in eq 2 above was used to calculate the lattice size *L*_c_ and *L*_a_ using 0.9 and 1.84 as K constants, respectively. It was found that AmSh waste AC had a higher crystalline size (*L*_c_), followed by AmWa waste and then AmSe waste ACs, as shown in Table 3.

**Figure 6:**
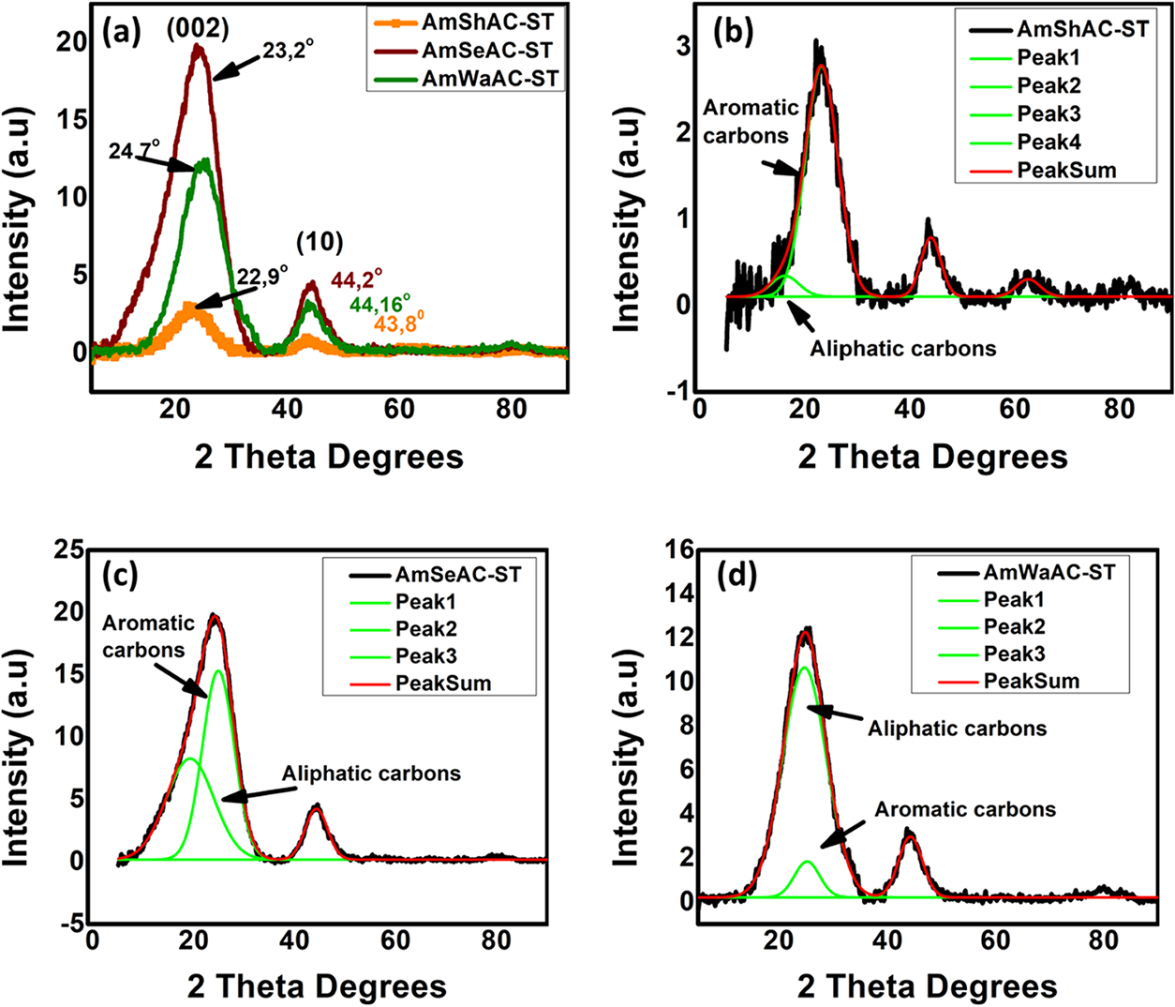
XRD patterns of Amarula waste-derived activated carbons: (a) diffraction patterns for all activated carbons and (b–d) deconvoluted (002) bands for AmShAC-ST, AmSeAC-ST, and AmWaAC-ST, respectively.

**Table 3:** Crystallinity Parameters of Amarula Waste ACs

	lattice size Ǻ				
AC type	*L*_c_	*L*_a_	*d*_002_ (Ǻ)	*I*_Π_/*I*_λ_	*f*_a_
AmShAC-ST	10.66	41.68	3.88	8.63	0.93
AmSeAC-ST	7.81	34.3	3.83	1.87	0.55
AmWaAC-ST	9.04	34.04	3.6	0.16	0.24

It was further found that the AmShAC was more graphitic because the interlayer *d*-spacing was higher, as shown in Table 3, and followed the trend AmShAC-ST > AmSeAC-ST > AmWaAC-ST.

Upon deconvolution in Figure 6b–d, it was observed that the 002 reflection basal planes possessed two bands (λ band & Π band). The first peaks (λ bands) on the left side at 2θ values of 16.1, 19.2, and 24.7°, were attributed to the aliphatic carbon chains for AmSh, AmSe, and AmWa waste ACs, respectively. The second peaks were due to the Π band arising from carbons in the aromatic rings^78^ at 2θ angles of 23.1, 24.8, and 21° respectively. The ratio of the carbon atoms in the aliphatic to that of the aromatic rings was determined using the integrated area under each fitted curve, using eq 7 ^78^

7 $$f_{a}=A_{\prod }/A_{\prod }+A_{\lambda }$$

where *f*_a_ is the aromaticity of AC, *A*_Π_ is the area of carbons in the aromatic rings, and *A*_λ_ is the area of the aliphatic carbon chains in ACs. The results in Table 3 showed that the aromaticity (*f*_a_) followed the trend AmShAC-ST > AmSeAC-ST > AmWaAC-ST.

#### 3.1.6 FTIR Analysis

The FTIR spectra of the processed Amarula waste in Figure 7 showed fewer vibrational bands compared to the unprocessed Amarula waste.^53^ The O–H stretching band, which was very broad at 3600–3100 cm^–1^, became very strong, highly intense, and less broad in the current study. This is because processed Amarula waste loses its hydrophilicity, meaning that less moisture is absorbed by the material. This is confirmed by the TGA analysis in Figure 3, where the first stage of decomposition, attributed to moisture loss, decreased due to a lack of moisture on the surface, resulting from the degradation of hemicellulose and cellulose. Therefore, the present O–H functional groups at 3442 cm^–1^ in the Amarula waste ACs spectra are due mainly to the phenols, lactones, and carboxylic groups.

**Figure 7:**
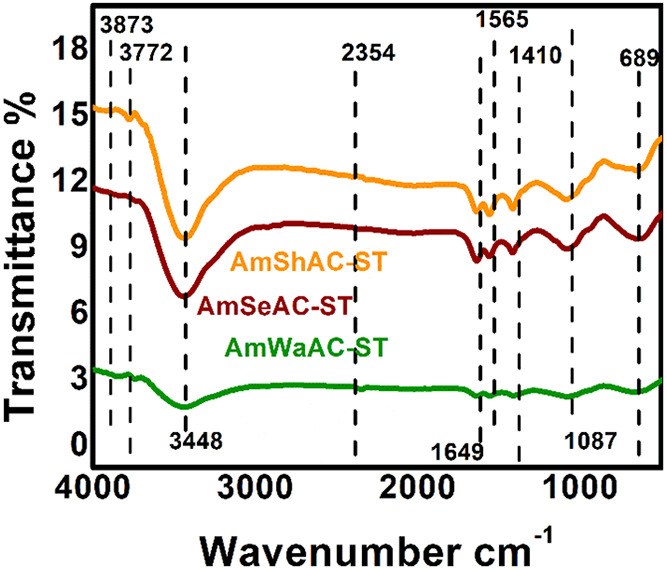
FTIR spectra of processed Amarula wastes.

More of these acidic groups were formed because steam was passed through during thermal processing. It was also observed that the two vibrational bands that were due to C–H symmetric and asymmetric bands of methyl and ethyl at the range 2850–2950 cm^–1^ in the unprocessed Amarula waste biomass^53^ have either now disappeared or are too tiny to be identified in the processed Amarula waste. This was attributed to the oxidation of the aliphatic carbon chains during steam activation and the establishment of more stable C=C and C≡C functional groups on the surface. Moreover, other new weak vibrational bands were observed in the range of 3700–3900 cm^–1^, which were attributed to the sp C–H of alkynes. These bands were confirmed by the C≡C functional group, which appeared as a weak band between 2300 and 2400 cm^–1^. This C≡C triple bond functional group was also observed by other researcher.^69,80,81^ The peak, which was due to a C=O vibrational band at 1737 cm^–1^ in the unprocessed Amarula waste,^53^ has now disappeared in the processed Amarula waste because of the thermal breakdown of the hemicellulose and has formed more carboxylic groups. The C=O stretch at 1649 cm^–1^ is attributed to the carbon–oxygen conjugation with carbon basal planes of carbonyl, quinols, lactose, and carboxylic groups in ACs.^18^ The respective C=C and C–C stretching bonds at 1565 and 1410 cm^–1^ confirm the vibrating bands in the condensed skeleton of aromatic rings in the Amarula waste ACs. It was further observed that the peak, which was very strong and highly intense at 1031 cm^–1^ in the unprocessed Amarula waste,^53^ has shifted to 1087 cm^–1^ and has become very weak and less intense in the processed Amarula waste. This is because of thermal processing, which broke down the C=O bonds of the present carbonyl functional groups and formed more of the C–O bonds for carboxylic, lactone, and phenolic groups, reducing the vibrational band of the C–O bonds. The sp^2^ C–H out-of-plane bond shifted from 897 to 659 cm^–1^ in the processed Amarula waste, confirming the formation of more aromatic rings. Other researchers^18,23^ also found similar FTIR bands of ACs as in this study. It can therefore be concluded that of the three Amarula waste ACs in the current study, the AmWaAC-ST had the strongest bonds of alkynes. This was due to the high content of volatiles and aliphatic carbon, as confirmed in Figure 3c of the TGA analysis and in Figure 6d of the deconvoluted peaks of XRD analysis, respectively. Despite that, it was found that the AmSh ACs had more of the hydroxyl groups and the carboxylic groups present because they had the strongest peaks at 3443 and 1087 cm^–1^, followed by the AmSe waste ACs and then the AmWa waste ACs.

#### 3.1.7 Textural Properties

Upon analysis of Amarula waste ACs produced at a 45 min steam residence time, it was found that the AmShAC had the highest BET surface area of 740 m^2^/g and the highest total pore volume of 0.37 cm^3^/g, followed by the AmWa waste, then the AmSe waste AC, as shown in Table 4. These results confirm the SEM analysis in Figure 4, where the three Amarula waste ACs had a morphology showing very large pores of different shapes for each AC type.

**Table 4:** Comparison of Textural Surface Analysis of Amarula Waste ACs with ACs from Literature^6,25,38,56,57,59,82–85^

	conditions			surface area				pore volume			pore size	
AC precusor	activation method	temparature (°C)	residence time (min)	BET S-area m^2^/g	t-micropore S-area (m^2^/g)	external S-area (m^2^/g)	BJH S-area (m^2^/g)	BJH pore vol (cm^3^/g)	t-micropore (cm^3^/g)	total (cm^3^/g)	average size (*n*)	BJH pore size (nm)
oats hulls	steam	800	120	625								
corn stove	steam	800	120	311								
corn corb	steam	850	60	607								
bamboo waste	steam	850	60	870								
guayule bugasse	steam	800	45	716								
apricot stones	steam	700	120	820					0.46	0.5		
almond shells	steam			484								
rubber seeds shells	steam			948								
Moso bamboo	steam	800	60	486	385						1.92	
Ma bamboo	steam	800	60	464	391						2.00	
wood chips birch	steam	800	240	617	318	300			0.136	0.509		
wood chips spruce	steam	800	240	679	317	369			0.136	0.555		
peat	steam	800	240	543						0.555		
commercial AC	unknown			541						0.18	0.33	
Amarula fruit waste	steam	800	45	425	357	68	38	0.0351	0.0187	0.23	2.20	3.60
Amarula seed waste	steam	800	45	279	238	41	19	0.01695	0.017	0.15	2.16	3.55
	steam	800	30	434	345	88	182	0.0923	0.1783	0.29	2.66	3.15
Amarula shell waste	steam	800	45	740	498	241	201	0.1749	0.1983	0.37	2.75	3.47
		800	75	881	557	324	312	0.2314	0.1998	0.78	2.89	3.85
		800	90	1193	404	789	905	0.3345	0.2023	0.98	3.27	4.40

The observed high-surface area and the increased porosity in the thermally processed Amarula waste are attributed to the removal of volatiles from the Amarula waste biomass precursors during the pyrolysis stage. This first stage formed mainly micropores and more carbon atoms, which contributed to the greater surface area and porosity of the intermediate product (biochar). The second stage, which involved applying steam to the biochar, created more pores, widened them, and further deepened them, depending on the precursor’s structure or the biochar’s structure. Similar results were also found by other researchers, who also produced ACs from various precursors using a steam-activation process.^6,82,86^ The textural properties of Amarula waste ACs in this study are listed in Table 4, where they were compared with other ACs reported by other researchers.^6,25,38,56,57,59,82–85^ Amarula waste ACs were found to be compatible with other ACs in the literature. The surface area of AmSh waste ACs was also compared with that of commercial AC, which was purchased from Sigma-Aldrich in South Africa. It was found that the AmShAC produced at 45 min had a higher BET surface area of 740 m^2^/g compared to commercial AC that had a BET surface area of 541 m^2^/g with a total pore volume of 0.42 cm^3^/g. This confirms that the AmSh ACs can be commercialized and competent in the AC industry. Besides that, the SEM morphology confirmed that Amarula shell AC had a glassy-like structure of carbon suitable for industrial purposes.

Textural properties were further studied for the selected AmShAC to assess the effect of the steam residence time. As the steam residence time increased from 30 to 90 min, the BET surface area increased from 434 to 1193 m^2^/g. It was also found that the porosity increased with an increase in the steam residence time, as shown in Table 4. This is attributed to steam activation removing sp^3^ bulk carbon; therefore, the longer the char is exposed to the steam, the more carbon is removed and more pores are formed, resulting in a higher surface area. More mesopores are formed due to the H_2_O molecule, which has easy access to the already available micropores on the solid. In addition, as the H_2_O molecule removes carbon atoms, it occupies the space occupied by carbon, creating more mesopores. Again, it is because the longer the material is exposed to steam, the more the entire surface of the material gets enough exposure for the reaction, consequently increasing the BET surface area. Longer exposure also produced more widened and deepened pores, leading to more mesopores and macropores being observed at 90 min residence time. Similar results to these in this study were obtained by other researchers who determined the effect of steam residence time on ACs.^25^

### 3.2 Desulphurization of ≈100 ppm Model Diesel Fuel

#### 3.2.1 Effect of Adsorption Time on Processed Amarula Waste

Figure 8 shows the effect of DBT adsorption time on adsorption efficiency using processed Amarula waste. All Amarula-derived ACs improved the adsorption of the DBT molecule in model diesel fuel compared to the unprocessed Amarula waste.^53^ This might be due to micropores and mesopores introduced by thermal processing and steam treatment, along with O-functional groups on the surface. Most of the DBT was removed within the first 60 min, reflecting the availability of accessible active sites on the surfaces of ACs. Beyond this point, the adsorption rate slowed until 180 min, as DBT molecules increasingly occupied the outer surface and had to diffuse deeper into micropores, where resistance and repulsion from previously adsorbed solute hindered uptake, hence the decrease in adsorption rate.^87^ A similar trend was observed in all of the Amarula waste Acs (Figure 8).

**Figure 8:**
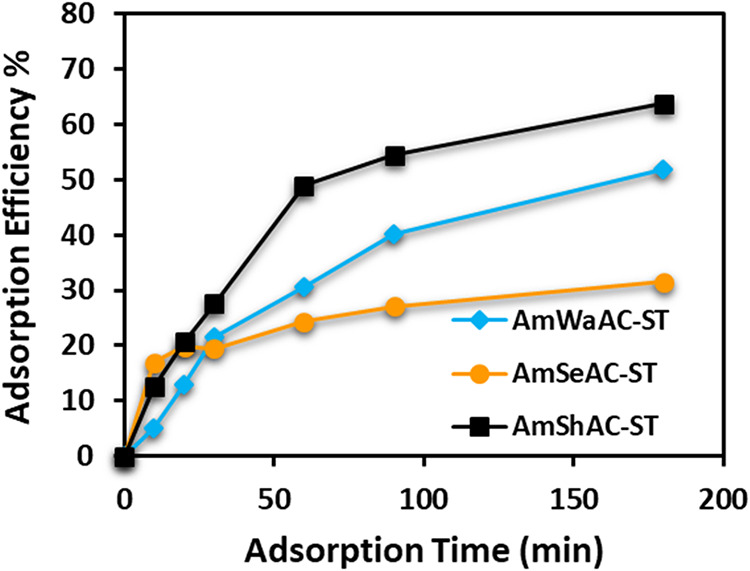
Effect of the adsorption time of DBT on Amarula waste ACs.

For AmSe-derived AC, the removal of the DBT molecule within the first 10 min was higher than that of the AmSh and AmWa ACs. However, from 30 to 180 min, the AmSeAC exhibited lower adsorption capacity. The initial rapid update might be due to its high adsorption affinity,^88^ resulting from interactions between the aromatic rings of ACs and the DBT molecule. Supporting this, Figure 6b shows that AmSeAC contained more aromatic carbons than the other two Amarula-derived ACs. The limited adsorption beyond the initial stage is likely due to its smallest t-micropore volume of (0.017 cm^3^/g, Table 4), which restricted further diffusion and uptake. This shows that the AC’s porosity plays an important role, a conclusion consistent with findings reported by other researchers.^89,90^

The AmSh waste AC was found to have higher activity from 30 to 180 min. This enhanced performance could be attributed to their greater microporosity, which facilitated the diffusion of DBT molecules into the micropores until saturation was reached. Similar observations were reported by Rajapaksha et al.^86^ who found that mesoporous biochar facilitated the adsorption of sulfamethazine. The presence of abundant mesopores in AmShAC was further confirmed by the textural analysis in Table 4. In addition to porosity, surface chemistry also played a role: FTIR showed a highly intense peak for O–H and C–O stretching, which was mainly due to carboxylic acids, which likely contributed to stronger adsorption through electrophilic addition, as oxygen atoms can form O–S bonds with sulfur species. Overall, the adsorption capacity of Amarula waste Acs followed the trend: AmShAC-ST > AmWaAC-ST > AmSeAC-ST within 30–180 min. It can thus be concluded that AmSeAC showed the fastest initial adsorption rate of DBT in model diesel within 10 min, while AmShAC sustained higher adsorption over longer times, with AmWaAC displaying intermediate behavior.

AmSh waste was selected and used to evaluate the effect of the steam residence time on DBT removal from model diesel fuel, as shown in Figure 9. It was found that the adsorption capacity increased with longer activation time of the ACs. Specifically, AmShAC-ST-45 min adsorbed 50% of DBT within 60 min, whereas AmShAC-ST-90 min adsorbed more than 50% of DBT molecules in model diesel within just 30 min, as shown in Figure 9; despite that, the adsorption quickly slowed down toward equilibrium. This enhanced performance is attributed to the greater development of micro-, meso-, and macropores in ACs produced over extended activation, as confirmed in Table 4. Furthermore, the FTIR spectra not presented here confirmed the intense, broader peak of C–O at 1087 cm^–1^, indicating abundant carboxylic groups. These improved textural and surface properties enabled more effective adsorption, thereby massively reducing the concentration of the organosulfur compounds.

**Figure 9:**
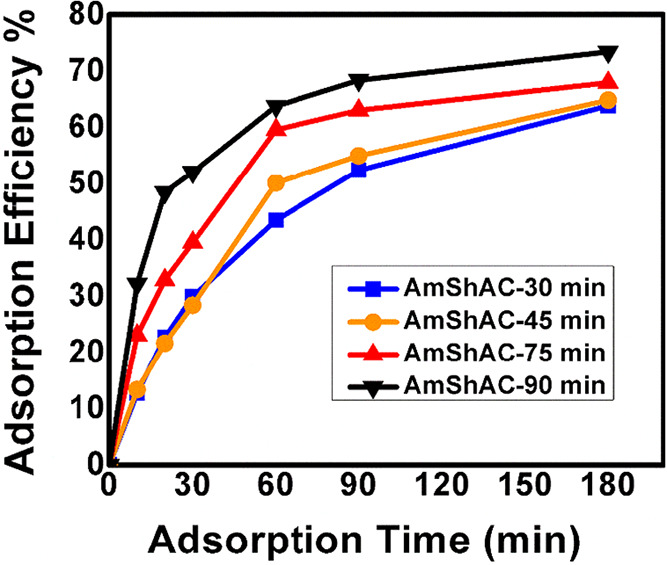
Adsorption efficiency of Amarula shell ACs produced at different steam residence times.

In summary, AmSeAC showed rapid initial DBT uptake due to its aromatic carbon content, but limited microporosity led to an early plateau. In contrast, AmShAC sustained higher adsorption through greater mesoporosity and abundant O–H and C–O groups, which enhanced interactions with sulfur compounds. Longer steam activation (90 min) further improved the pore development and functional group intensity, yielding faster and more efficient DBT removal. Overall, AmShAC outperformed other waste-derived carbons, offering mechanistic insights and underscoring its potential for cleaner fuel applications.

#### 3.2.2 Kinetic Models

Kinetic models were applied to the experimental data for the processed Amarula waste ACs, as shown in Figure 10. Equations 8–10 were used to determine the best fit of the model

8 $$\ln\left(q_{e}-q_{t}\right)=\ln\left(q_{e}\right)-k_{1}t$$

9 $$t/q_{t}=1/k_{2}{q_{e}}^{2}+\left(1/q_{e}\right)t$$

$$q_{e}=k_{i}\,t^{1/2}+C$$ 10

at which *q_t_* and *q_e_* (in mg/g) were the adsorption capacity at time (*t*) and at equilibrium (*e*). The *C* represents the intercept, which describes the thickness of the boundary layer on the surface of the adsorbent, whereas *k*_1_, *k*_2_, and *k*_i_ are kinetic constants in 1/mg, mg/g·min, and mg/g·mg^1/2^ for pseudo-first-order, pseudo-second-order, and intraparticle diffusion models, respectively.^81,91,92^ According to literature, when a correlation value (*R* ^2^) is close to unity (1), then the fitted results follow that model.^72,93,94^

**Figure 10:**
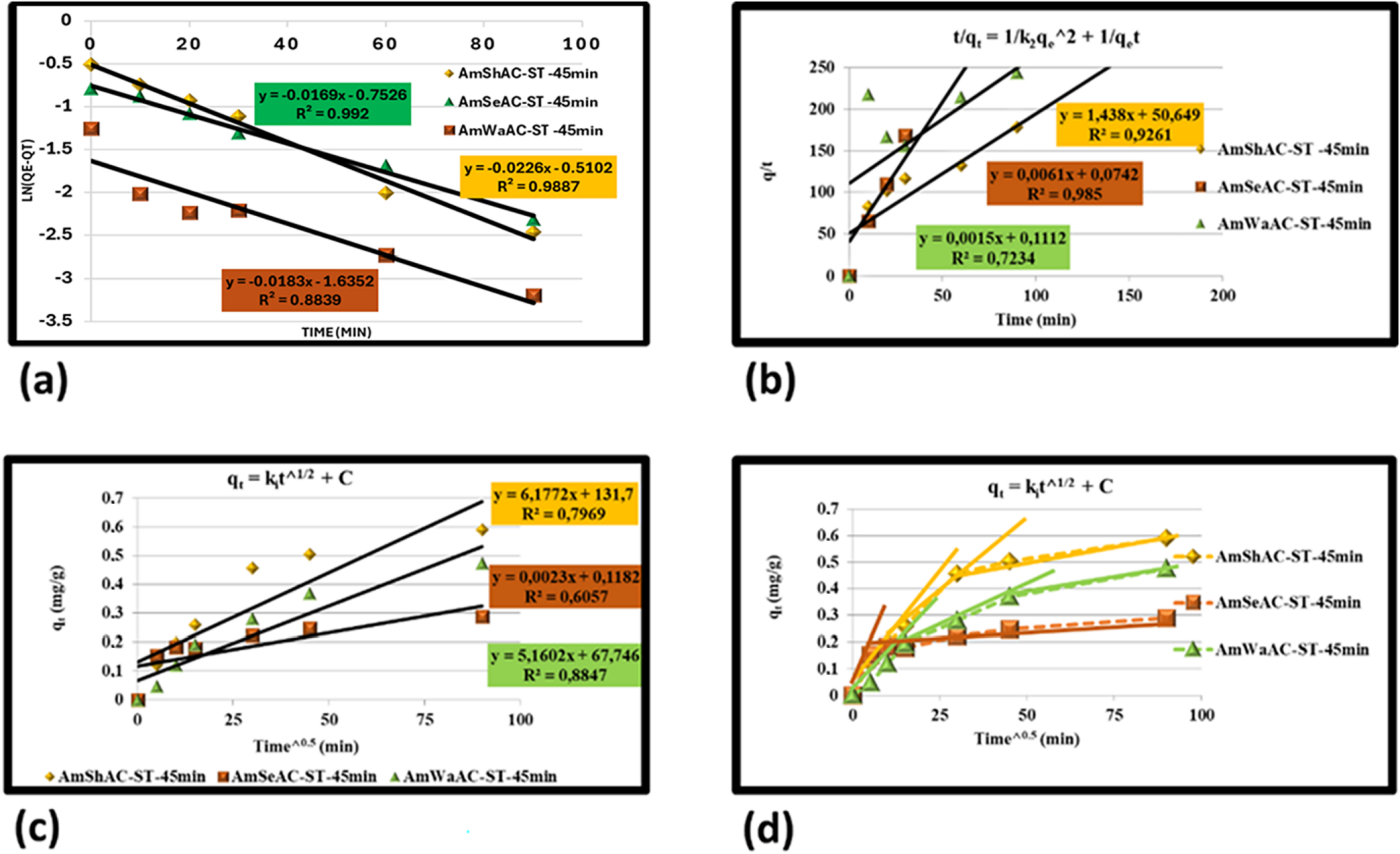
Kinetic models of (a) pseudo-first-order, (b) pseudo-second-order, (c) intraparticle diffusion, and (d) intraparticle diffusion, showing multilinear steps of adsorption.

This study showed that AmShAC-ST and AmSeAC-ST followed both the pseudo-first-order and the pseudo-second-order kinetic models, as indicated by *R* ^2^ values close to unity (above 0.9), as shown in Table 5. This implies that both chemisorption and physisorption may have been involved in the adsorption of DBT. Conversely, the AmWaAC-ST had the highest *R* ^2^ value for intraparticle diffusion (0.8847), comparable to that for pseudo-second-order kinetics (0.8839). This indicates that adsorption on AmWaAC-ST may have occurred primarily via particle diffusion and physisorption.

**Table 5:** Kinetic Parameters of Amarula Waste ACs

	Pseudo first order		Pseudo second order				intraparticle diffusion				
ads type	*q*_*e* calc_ (mg/g)	*k*_1_ (I/mg)	*R* ^2^	*q*_*e* calc_ (mg/g)	*k*_2_ (mg/g·min)	*R* ^2^	*q*_*e* calc_ (mg/g)	*k*_i_ (mg/g·mg^0.5^)	*C*	*R* ^2^	*q*_*e* exp_
AmShAC-ST	850.394	–3.40 × 10^–02^	0.988	0.695	1.05E + 0.2	0.926	687.650	6.1772	131.700	0.7969	0.5905
AmSeAC-ST	133.807	–2.56 × 10^–02^	0.992	163.934	2.76 × 10^–06^	0.985	0.330	0.0023	0.118	0.6054	0.2884
AmWaAC-ST	0.28078	2.80 × 10^–03^	0.883	666.667	2.50 × 10^–07^	0.723	532.160	5.1602	67.746	0.8847	0.4733

Based on the structure of Amarula ACs, adsorption may occur via physisorption when the organosulfur molecules are adsorbed inside the pores of ACs and when the aromatic rings form a pi-pi complex interaction with the aromatic rings of the DBT molecule. However, limited chemisorption could also occur through stronger interactions involving surface oxygen functionalities (e.g., C–O, C=O) that can form electrophilic bonds with sulfur atoms, thereby enhancing desulphurization performance.

Furthermore, this study used Chi-square (χ^2^) to validate the best-fit kinetic models obtained for the results. Chi-squared (χ^2^) test is normally analyzed under the same abscissa and ordinate to determine the best-fit model, which makes it more advantageous. It states that if the calculated Chi-squared value (χ^2^) is smaller for a model, then the results follow that model. The χ^2^ results for the adsorption of DBT in model diesel on processed Amarula waste samples, seen in Table 6, were obtained using eq 11, where *q*_*e* exp_ and *q*_*e* calc_ are the equilibrium adsorption capacity for experimental and calculated data, respectively. Therefore, the smallest χ^2^ values were found to be 0.019 for AmShAC-ST under the pseudo-second-order model, 0.0047 for AmSeAC-ST under the intraparticle diffusion model, and 0.078 for AmWaAC-ST under the pseudo-first-order kinetic model. These χ^2^ results are consistent with the results in Table 5 in terms of comparison of *q*_*e* calc_ and *q*_*e* exp_

11 $$X^{2}=\sum {\left(q_{e\;\exp}-q_{e\;\mathrm{calc}}\right)}^{2}/{q_{e}}_{\mathrm{calc}}$$

**Table 6:** Chi-Squared Values for Amarula Waste ACs

AC type	Pseudo 1st order	Pseudo 2nd order	intraparticle diffusion
AmShAC-ST-45 min	1.22E + 06	1.86 × 10^–02^	7.99E + 05
AmSeAC-ST-45 min	6.16E + 04	9.29E + 04	0.0047
AmWaAC-ST-45 min	7.80 × 10^–02^	9.38E + 05	5.97 + 05

For this reason, it can be concluded that the adsorption mechanism of AmSh waste AC occurred mainly via chemisorption, in which there was involvement of valence forces through the exchange or sharing of electrons between the outer surface of AmShAC and the DBT molecules in model diesel fuel. The AmSeAC, however, followed intraparticle diffusion, suggesting that molecules continued to be adsorbed from the outer surface into the adsorbent’s pores. On the other hand, the AmWaAC followed mainly a pseudo-first-order model based on χ^2^ values, but there might also have been intraparticle diffusion, as indicated by its *R* ^2^ values, which were highest.

Although the χ^2^ values of the Amarula waste ACs showed consistency of *q_e_* values for calculated and experimental data, the graph in Figure 10c showed that the plots did not pass through the origin. Therefore, the intraparticle diffusion model is not the sole rate-limiting step for the adsorption mechanism; other adsorption mechanisms are involved on the surface.^95^ The intercept *C*, which is referred to as a boundary layer effect, showed a value of 132 for AmShAC that was higher than that for AmWaAC, confirming that there was a greater contribution of surface adsorption in the rate-limiting step. Conversely, the same graph extended in Figure 10d also showed multilinear plots for Amarula waste ACs, further confirming different adsorption mechanisms. This was attributed to differences in the mass-transfer rates between the initial and final adsorption stages. In the initial, fast stage, adsorption occurred via film diffusion, whereas in the second stage, it occurred via pore diffusion and intraparticle transport.^95^

### 3.3 Desulphurization of Conventional Diesel Fuel

Conventional diesel containing approximately 280 ppm sulfur was subjected to the same conditions as the model fuel in the adsorptive desulfurization experiments using AmShAC-ST. As shown in Figure 11a,b, the FID chromatogram confirmed that the diesel comprised numerous organic compounds. The PFPD analysis further revealed multiple sulfur-containing species, most of which were heavier and more complex than DBT. The DBT peak appeared at a retention time of 14.7 min (not shown).

**Figure 11:**
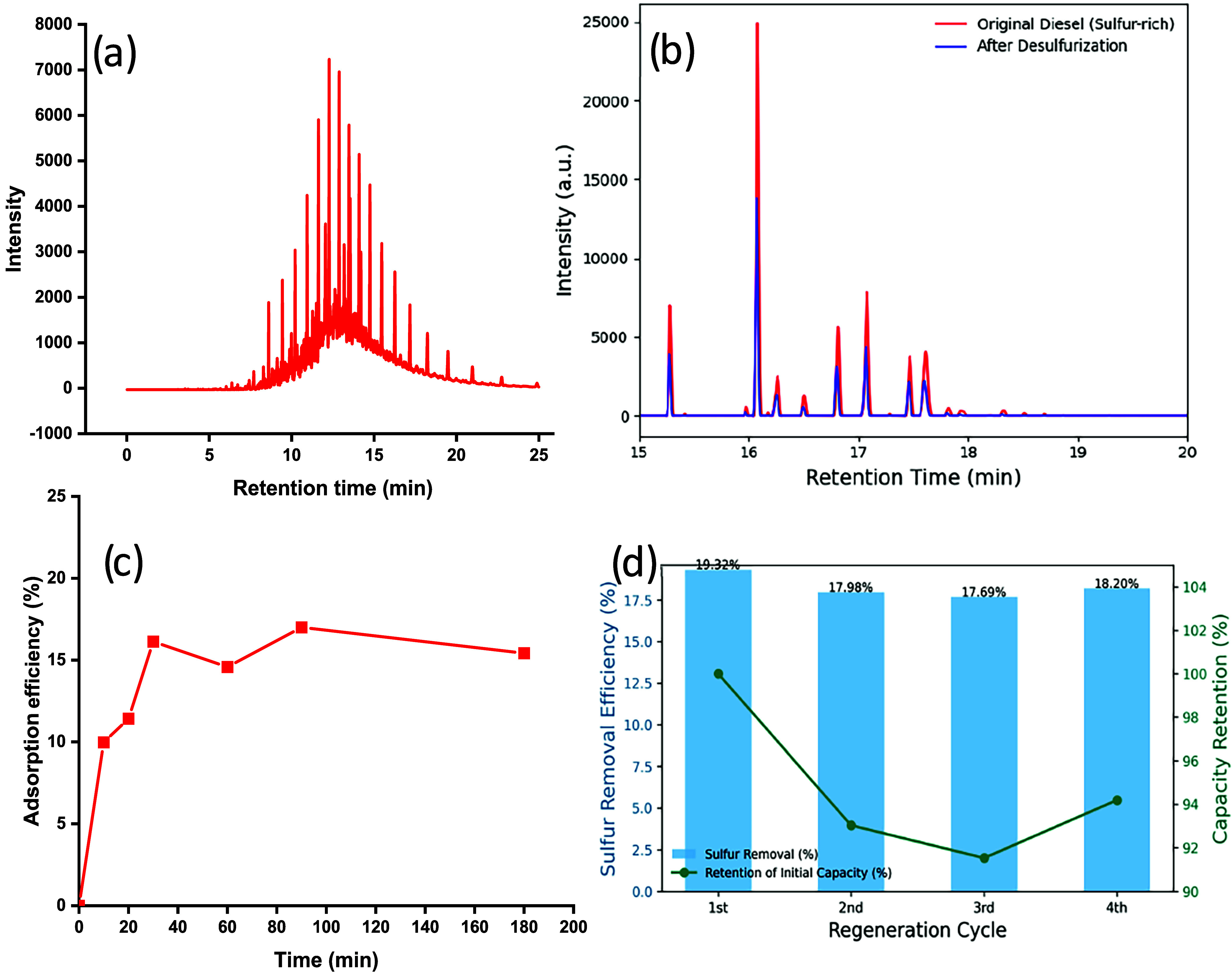
(a) FID chromatogram for real diesel, (b) PFPD chromatogram sulfur distribution, (c) effect of adsorption time on conventional diesel, and (d) regenerability of AmShAC-ST using conventional diesel.

The adsorption process occurred predominantly within the first 30 min, after which no significant additional sulfur uptake was observed, Figure 11c. Compared with the model fuel, the sulfur removal efficiency dropped markedly, from about 70% to just about 20%, indicating a loss in adsorption capacity. This decline is attributed to competitive adsorption among the numerous hydrocarbon species present in the real diesel, as evidenced by the complex FID profile (Figure 11a). The presence of aromatics and polar compounds likely hindered access to adsorption sites, reflecting the limited selectivity of activated carbon. Future improvements should therefore aim to enhance adsorbent selectivity toward sulfur compounds, e.g., surface functionalization.^96,97^

Interestingly, the extent of sulfur removal correlated directly with the sulfur concentration (Figure 11b), with no clear dependence on other physical properties such as molecular weight. This behavior suggests that the process was primarily governed by physical adsorption rather than chemical bonding.

Finally, the adsorbent’s regenerability was examined using real diesel. Although its activity was significantly lower than that observed with the model fuel, the material retained steady desulphurization performance after four consecutive adsorption regeneration cycles (Figure 11d). This demonstrates reasonable structural stability and reusability, even under the more complex chemical environment of real diesel fuel.

## 4 Conclusions

This study demonstrates that the Amarula production wastes were successfully converted into ACs through pyrolysis and steam activation. The AmSh, AmSe, and AmWa feedstocks were thermally processed at 800 °C, and the effect of the steam-activation residence time was investigated. The surface area, total pore volume, and mesoporous pore volume of AmShAC increased with the residence time, attributed to the reaction of steam with fixed carbon.

TGA/DTG revealed that the AmSh-derived ACs were more thermally stable than those derived from AmSe and AmWa. The produced ACs showed textural differences comparable to commercial activated carbon, with BET surface areas of 740, 430, and 240 m^2^/g for AmShAC-ST, AmWaAC-ST, and AmSeAC-ST, respectively. FTIR spectra confirmed the presence of abundant oxygen-containing functional groups on the acidic surfaces of the ACs, which contributed to adsorption performance by facilitating interactions with sulfur compounds. The deconvoluted XRD patterns indicated a higher degree of aromatic carbon ordering in AmShAC, making it more crystalline than AmWaAC and AmSeAC. These structural features collectively explain the superior adsorption behavior of AmShAC.

The desulfurization performance of the produced ACs was evaluated by using dibenzothiophene (DBT) as a model compound. AmShAC achieved 64% DBT removal, outperforming AmWaAC (54%) and AmSeAC (31%). When steam activation was extended to 90 min, AmShAC reached a surface area of approximately 1200 m^2^/g and a pore volume of 0.40 cm^3^/g, resulting in 73% DBT removal. This efficiency was comparable to commercial AC (72%), despite differences in pore structure and surface area. These findings confirm that Amarula waste-derived ACs can match the performance of conventional adsorbents.

However, when applied to real diesel fuel, AmShAC showed a significant drop in activity to about 20%. This reduction was attributed to competitive adsorption of sulfur compounds with other hydrocarbons present in the complex fuel matrix. While this highlights a limitation in selectivity, it also points to opportunities for further materials tuning, such as tailoring pore size distribution, modifying surface chemistry, or introducing catalytic functionalities, which could enhance selectivity toward sulfur species in real fuels.

Overall, the Amarula waste-derived activated carbons demonstrate strong potential for scale-up to commercial production if quality control is maintained. Significantly, these developed ACs have the potential to effectively reduce DBT content in diesel fuel if material selectivity is improved, thereby mitigating harmful emissions and supporting cleaner fuel technologies. Future work should focus on improving selectivity in complex fuel systems, benchmarking performance across diverse sulfur species, and optimizing activation processes for industrial viability.
